# Time to the first relapse after the transcranial magnetic stimulation with H7-coil in patients with predominant negative symptoms of schizophrenia; A randomized, sham controlled trial

**DOI:** 10.1192/j.eurpsy.2025.2237

**Published:** 2025-08-26

**Authors:** K. Matić, I. Š. Filipčić, Ž. Milovac, T. Gajšak, K. Bosak, I. Orgulan, V. Požgaj, Ž. Bajić, V. Grošić, I. Filipčić

**Affiliations:** 1 Psychiatric Clinic Sveti Ivan; 2 University Hospital Cnetre Zagreb, Zagreb; 3 Faculty of Dental Medicine and Health, Josip Juraj Strossmayer University of Osijek, Osijek, Croatia

## Abstract

**Introduction:**

Patients with predominant negative symptoms of schizophrenia experience severe functional impairment and limited response to pharmacological treatments. Transcranial magnetic stimulation (TMS) has shown potential for treating negative symptoms, but its impact on long-term outcomes, such as time to relapse, remains underexplored.

**Objectives:**

This study aimed to determine whether TMS with an H7-coil prolongs time to first relapse compared to sham stimulation in patients with low positive symptomatology and predominant negative symptoms.

**Methods:**

This study was a randomized, sham-controlled trial at the Psychiatric Clinic Sveti Ivan, Zagreb, Croatia. The target population were patients with PANSS negative symptoms score > 24 and PANSS positive symptoms score < 20, on stable pharmacotherapy for at least three months The intervention group received high-frequency TMS with an H7-coil, while the control group received sham stimulation, both applied once daily for 20 sessions over four weeks. The outcome was time to first psychiatric relapse, defined as psychiatric rehospitalization. Kaplan-Meier survival curves, log-rank tests, and Cox proportional hazards models were used for statistical comparisons.

**Results:**

A total of 76 outpatients with schizophrenia, aged 18-55 were included; 33% were women. Over the 12-month follow-up, 29% in the H7 group and 24% in the sham group experienced a relapse. The median time to relapse was not reached in either group. The hazard ratio (HR) for relapse in the H7 group relative to sham was 0.82 (95% CI 0.34; 1.97), suggesting no significant effect of TMS on delaying relapse. Adjusted Cox regression model for age, gender, baseline severity of negative and positive symptoms, pharmacotherapy, and number of prior hospitalizations showed similar results (HR = 0.85, 95% CI 0.30; 2.46, p = 0.769). Significant predictors of relapse were baseline severity of negative symptoms (HR = 0.88, 95% CI 0.79; 0.99, p = 0.026) and the number of prior hospitalizations (HR = 1.81, 95% CI 1.16; 2.82, p = 0.009).

**Conclusions:**

No significant effect of H7-coil TMS on delaying relapse was observed in patients with schizophrenia and predominant negative symptoms. Median survival was not reached in either group, suggesting the need for longer follow-up to fully evaluate potential benefits. Baseline severity of negative symptoms and prior hospitalizations should be considered when assessing relapse risk in this patient population.

**Disclosure of Interest:**

None Declared

